# Frequency domain manipulation of multiple copy-move forgery in digital image forensics

**DOI:** 10.1371/journal.pone.0327586

**Published:** 2025-07-17

**Authors:** Tanzeela Qazi, Mohsin Shah, Mushtaq Ali, Faqir Gul, Muneer Ahmad, Ajmal Khan

**Affiliations:** 1 Department of Computer Science and Information Technology, Hazara University Mansehra, Pakistan; 2 Department of Computer Engineering, Gachon University, Seongnam-si, Gyeonggi-do, Republic of Korea; 3 Department of Computer Science, University of Roehampton London; 4 Communication and Information Research Centre, Sultan Qaboos University, Seeb, OMAN; National Textile University, PAKISTAN

## Abstract

Copy move forgery is a type of image forgery in which a portion of the original image is copied and pasted in a new location on the same image. The consistent illumination and noise pattern make this kind of forgery more difficult to detect. In copy-move forgery detection, conventional approaches are generally effective at identifying simple multiple copy-move forgeries. However, the conventional approaches and deep learning approaches often fall short in detecting multiple forgeries when transformations are applied to the copied regions. Motivated from these findings, a transform domain method for generating and analyzing multiple copy-move forgeries is proposed in this paper. This method utilizes the discrete wavelet transform (DWT) to decompose the original and patch image into approximate (low frequency) and detail coefficients (high frequency). The patch image approximate and details coefficients are inserted into the corresponding positions of the original image wavelet coefficients. The inverse DWT (IDWT) reconstructs the processed image planes after modification which simulates the multiple copy move forgery. In addition, this approach is tested by resizing the region of interest with varying patch sizes resulting in an interesting set of outcomes when evaluated against existing state-of-the-art techniques. This evaluation allows us to identify gaps in existing approaches and suggest improvements for creating more robust detection techniques for multiple copy-move forgeries.

## 1. Introduction

In the realm of image manipulation, encompassing tasks like composition, editing, tampering, forgery, or falsification, the primary casualty is the integrity and genuineness of the image. The usage spectrum spans from aesthetic enhancements at one end to malicious objectives, such as blackmail and character assassination, at the other. Widely accessible software tools like Adobe Photoshop, GIMP, or even XnView have exacerbated the situation. Irrespective of the virtuous intentions behind any innovation aimed at manipulating images, the potential for negative consequences remains significant. The responsibility of addressing such negative implications falls upon the forensic analyst. Whereas Presenting forgery in positive aspect is to focus on the technological advancement and skills involved in detecting it. The challenge of forgery compels the researchers to develop detection techniques that can enhance security and maintain the integrity of digital images. Described aptly as an ongoing “arms race” in [1], the competition between the manipulator and forensic analyst appears to have no foreseeable conclusion.

**Fig 1:**
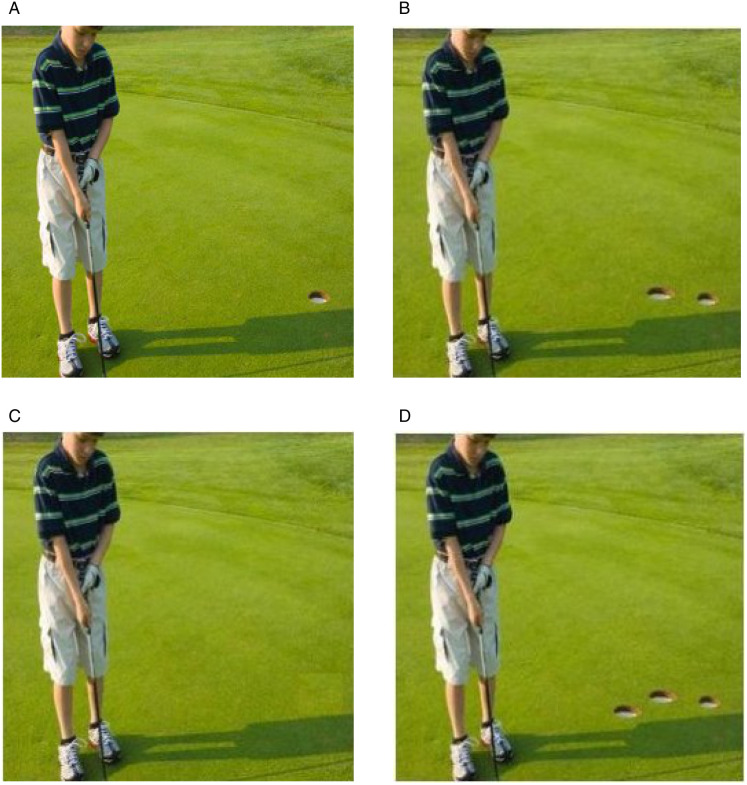
Copy Move Forgery Examples. (a) Original image, reproduced with permission from CASIA dataset (b) Copy move forgery (c) Copy move forgery hiding objects (d) Multiple copy move forgery, (b-d) Copy-move forgeries generated by our proposed method.

The digital image forensics deals with image fakery by examining the veracity of digital images. Authenticity and trustworthiness of images are both legally and socially very important. For security purposes, several approaches have been developed, broadly categorized as active and passive methods. Research in passive methods has been getting increasing attention in the community because of the limitations in the active counterparts, especially its reliance on watermarks; it should be embedded at the time of image acquisition that requires a specially equipped camera or devices and, secondly, most of the watermarks degrade the image quality while manipulating the image for the insertion of watermark or related processing [2–4]. The passive approaches or digital image forensics are classified as, image splicing, image retouching and copy move. Image splicing involves combining parts or two or more images to create a single composite image and image retouching refers to alteration of image to enhance or modify its visual appearance like contrast adjustment and smoothing or blurring objects [5,6]. In copy move forgery the region of an image is copied and pasted elsewhere in the same image on one or many locations demonstrated in Fig 1(b) and Fig 1(d) [2,7]. It is often used to hide the object to make it a part of original scene as shown in Fig 1(c).

Copy move forgery detection has been widely researched and categorized as conventional and deep learning approaches [7,8]. Several conventional approaches can affectively detect single copy move forgeries even though the copied region is passed through the geometrical transformation like rotation and scaling, noise addition and JPEG compression. However, when the image is forged in transform domain, these methods fail to produce satisfactory results for single copy move forgery [3]. For multiple copy move forgery, there is gap in deep learning approaches to detect multiple forged regions [4,5]. When image is passed through the transform domain for multiple

copy move forgery and is tested against the two state-of-art conventional approaches, however the result is unsatisfactory [9,10]. As multiple copy move plays an important role in making complex and misleading changes in digital images and the gap in existing detection approaches is motivated by the necessity to develop transformed multiple copy move forgery. A challenging multiple copy move forgery motivates our work to develop detection technique that can identify the complex and subtle manipulations.

In [3], single-region copy-move forgery in transformed domain is presented where both the patch and the host image undergo DWT at the same level. Each sub-band of the patch is then inserted into the corresponding sub-band of the host image at the specified location. Finally, the manipulated host sub-bands are processed using inverse DWT to produce the single copy move forged image. However, the literature emphasizes the necessity of advancing towards multiple transformed forgery detection because existing approaches fall short in identifying such manipulations, as demonstrated in [3]. This shift is crucial due to the notable gap in existing digital forgery detection methods within the domain of deep learning and conventional approaches.

In this paper, a multiple transformed copy-move image forgery method is presented that exploits the localized properties of the Discrete Wavelet transform (DWT). This method involves to crop a small region as a patch from the original image and this patch is pasted at specified different locations within the same original image known as host image. In preprocessing, this patch and host is converted from RGB to YCbCr color space and each component is processed through a single level Discrete Wavelet transform, resulting in a four sub-bands per component with its Approximate, vertical, horizontal and diagonal coefficients. The corresponding patch sub-bands were pasted onto the host sub-bands on the specified different locations, and inverse DWT was applied to produce the manipulated Y, Cb, and Cr components. After combining these components YCbCr image is obtained and then converted back to the RGB image. Furthermore, we generate instances of multiple copy-move forgery using varying patch image sizes to augment the complexity of the task and enhance the efficacy of current forgery detection methodologies. This approach aims to refine detection capabilities by presenting more challenging scenarios for analysis.

The remaining sections of the paper are organized as follows. Section 2 provides a brief overview of the relevant literature. Following this, Section 3 introduces the proposed method. Detailed simulation results are depicted in Section 4. The paper concludes with Section 5.

## 2. Related work

In this section, we explore the realm of image manipulation and the associated methodologies for identifying such distortions. The conventional approaches can easily detect the simple copy move forgery but when image is forged in transformed domain then existing state-of- art methods cannot detect these forgeries. The principal aim of our investigation was to create a manipulated image in transformed domain without readily noticeable visual cues or traces of tampering. Subsequently, we assess the robustness of the manipulated image generated using our proposed technique by comparing it against established state-of-the-art approaches.

Passive or blind methodologies necessitate no prior information about the original image, leaving the analyst solely with the final output [2]. In copy move scenario, the patch is derived from the host image, making detection challenging due to the identical nature of the source and destination images, with consistent color and noise throughout the rest of the image [2,3]. Image splicing involves a patch sourced from an image different from the host, making detection relatively straightforward as the source and destination originate from distinct images or sets of images [1,2]. Image retouching encompassing a diverse range of techniques can enhance the visual appeal of the image, this is considered the least malicious type of forgery and is extensively employed by editors in magazine photography [2,3].

Apart from image manipulation facilitated by readily accessible software, the researchers in [11] introduced a forgery technique for an experimental investigation on detection methods, demonstrating robust resistance against forgery detection. In this approach, the patch image mask is generated from the host image and subsequently applied to the host image to generate a forged image.

In [12], the procedure for image forgery involves selecting a region of interest (ROI), manipulating and combining image fragments, and applying necessary postprocessing to the final image. Typically, the process starts with extracting fragments and integrating transformed fragments into a new image using techniques like matting or pasting to create a visually cohesive composition. The claimed advantage of this method is its ability to produce an outcome without noticeable traces of tampering when exposed to existing forgery detection techniques.

**Fig 2:**
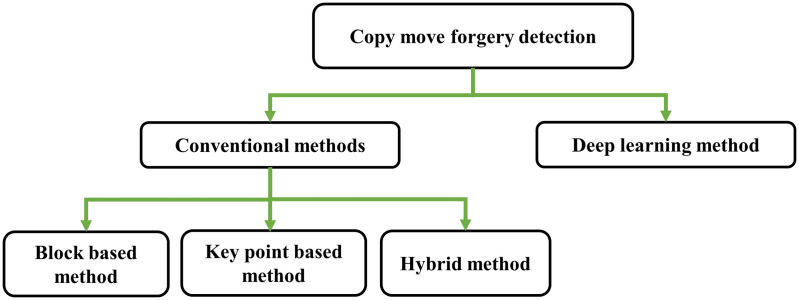
Classification of copy move forgery detection techniques.

In [13], various technologies and tampering methods were outlined for digital images that pose challenges in detection. According to the authors, verifying the authenticity and reality of digital photography has become increasingly intricate and demanding. Within the literature, numerous approaches exist; broadly classified in conventional and deep learning approaches as shown in Fig 2. Conventional approaches consist of block based, key point and hybrid approaches [14]. Block based approaches are highly desirable for identifying forged regions in copy-move forgery, as they perform matching in a block-wise manner, but these methods also have some imperfections and limitations, Firstly, these methods demonstrate low accuracy when subjected to post-processing operations such as adding noise, object blurring, compression, contrast alteration, or a combination thereof. Secondly, they are not resilient to rotation and scaling. Additionally, these techniques incur high computational costs [15].

Key point approaches are well suited to take fast decisions about the investigated image and have low computational cost and notable performance with respect to memory requirement and are appropriate for the detection of multiple forged regions. But these methods are not appropriate where the intensity values are uniform or regions are smooth and higher rate of falsely detected areas in images with natural similarity. These methods exhibit strong performance to post-processing attacks, rotation and scaling [16, 9, 10, 15]. An approach that combines the principles of block based and key point-based methods, as described in reference [17], is known as a hybrid approach. Some techniques are less effective in detecting multiple forgery attacks while others are proficient.

In 2020, a technique was introduced to identify copy-move forgery in images, employing PCET-SVD and Histogram of Block Similarity Measures (HBSM). PCET-SVD is utilized for feature extraction, while HBSM measures block similarity. While effective in detecting copy-move forgeries with geometrical transformations, this method exhibits weaknesses when faced with noise attacks and performs inadequately for small or multiple tampered regions [6].

In [5], a study presents an effective approach for copy-move forgery detection using deep learning-based feature extraction and matching. The process involves segmenting the image with SLIC (simple linear iterative clustering) method and employing VGG net for multi-scale feature extraction. An adaptive patch matching method is then used for forgery detection, reducing computational time and enabling the identification of rotated, scaled, and noisy images. Despite these strengths, the method falls short in detecting multiple forgery attacks.

The approach outlined in [18], the investigated image is divided into fixed-size overlapping blocks and each block undergoes the Tetrolet transform to extract features. The blocks are then lexicographically sorted based on four features and similarity is evaluated using a threshold. It demonstrates resilience against both small-scale and multiple copy-move forgeries, exhibiting superior time complexity. Nevertheless, a scaling tradeoff exists with optimal performance.

In the year 2021, a method was introduced to identify multiple copy-move forgeries in images, demonstrating robustness and efficacy. This approach entails the extraction of key point features utilizing GFAST (generic features from accelerated segment test) through adaptive thresholding and non-maxima suppression. Subsequently, Kd tree is employed for false match detection using RANSAC and ZNCC algorithms. While this technique proves effective for both simple and multiple forgeries, it remains untested against geometric transformations [16].

In [19], author introduces a method called QDL-CMFD, which employs a deep learning approach independent of image quality to detect forgery. QDL-CMFD utilizes generative adversarial networks for image enhancement and convolutional neural networks (CNN) for identifying forgery. It introduces a specialized dual-branch CNN architecture comprising two subnetworks: a manipulation detection subnetwork and a similarity detection subnetwork. Unlike many existing methods, QDL-CMFD can simultaneously detect multiple forged areas and determine both the source and target of the forgery. Furthermore, it demonstrates robustness against various pre-processing and post-processing attacks. QDL-CMFD exhibits excellent performance in detecting low-quality forged images and small areas.

In literature, from various studies on the subject of multiple image forgery, we’ve learned about different methods and challenges in this area and enhancing our ability to modify multiple image forgeries.

## 3. Transformed multiple copy move forgery

**Fig 3:**
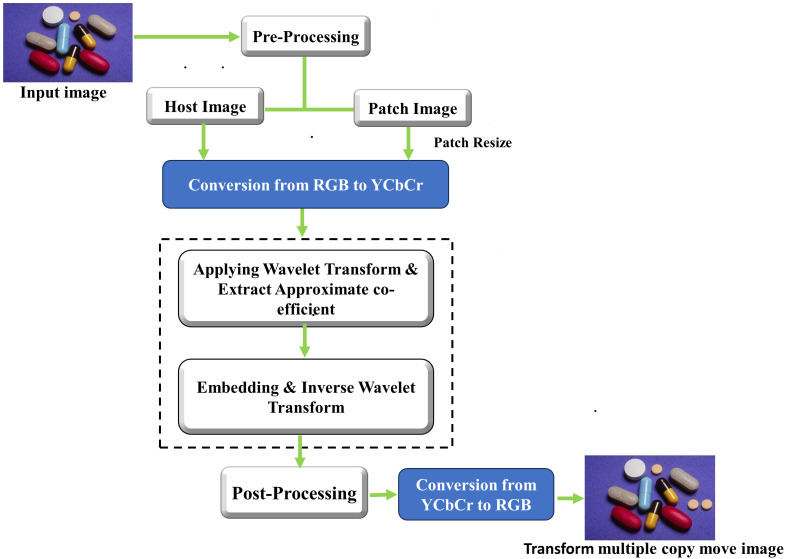
Flowchart of Transformed multiple copy-move forgery, The input image reproduced with permission from CASIA dataset and Transformed copy move forged image is generated by our method.

The flow diagram of our proposed transformed copy-move method is illustrated in Fig 3. In the pre-processing step, the image is converted to YCbCr format to separate luminance (Y) from chrominance (Cb and Cr) because brightness and colour can be processed separately allowing for more efficient processing. A frequency domain transformation is then applied, the 2D Discrete Wavelet Transform (DWT) using the Haar wavelet is first applied to all bands of the original image and a patch image. This decomposition breaks the both images into four sub-bands that is approximate coeffients (LL), horizontal coefficient (LH), vertical coefficient (HL), and diagonal coefficients (HH). The Haar wavelet, known for its simplicity and efficiency to find both low-frequency (approximation) and high-frequency (detail) components of the image. After this decomposition, the Haar wavelet coefficients from the patch image are embedded into the corresponding Haar wavelet coefficients of the original image at a specified location, simulating a transformed multiple copy-move forgery. Once the embedding is completed, the Inverse Discrete Wavelet Transform (IDWT) using the Haar wavelet is applied to reconstruct all Y, Cb, and Cr bands. The use of the Haar wavelet enables localized modifications in frequency domain to make it ideal for detecting subtle alterations like copy-move forgeries. In the post-processing, the transformed Y, Cb, and Cr sub bands are combined and converted back to the RGB format.

### 3.1 Pre-processing

Start with the input host or original image. Some preliminary pre-processing steps are necessary to process the image in the frequency domain. This includes

a) **Cropping Patches:**

**Fig 4:**
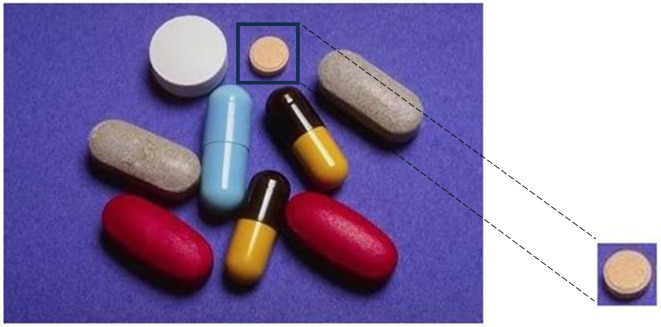
Original image, reproduced with permission from CASIA dataset and patch image is generated by our method.

Cropping the area of interest from the original or host image *I*_*o*_, which is referred to as the patch image *I*_*p*_ as in Fig 4(a).

b) **Resize:**

**Fig 5:**
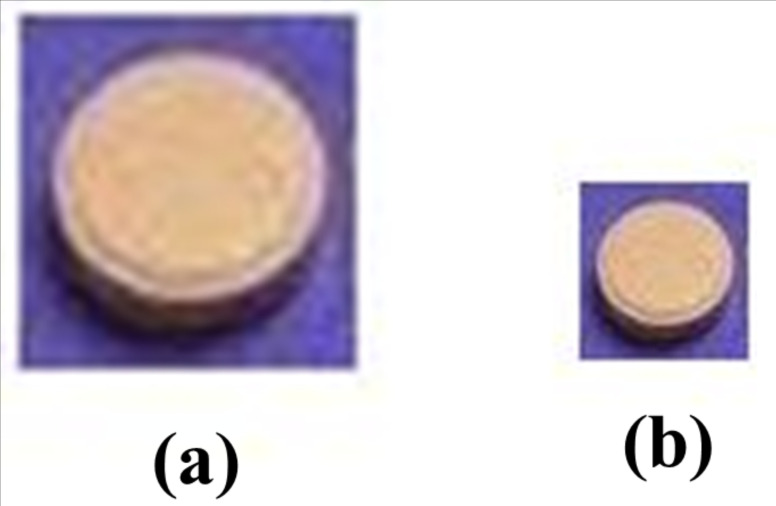
Patch images (a) Patch image size 1, (b) Patch image size 2, (a) and (b) Images are generated by our method.

When dealing with multiple forged regions, the size of the patch image can be adjusted during this step as in Fig 5(a) and Fig 5(b). Resizing the patch image can introduce additional challenges.

c) **Convert to YCbCr:**

**Fig 6:**
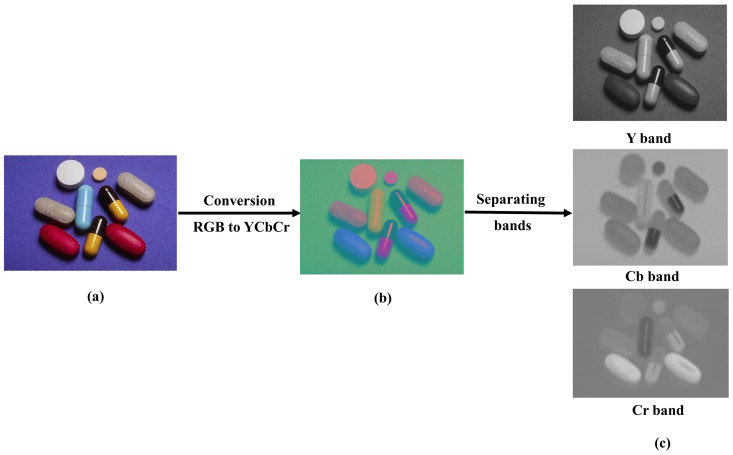
Image preprocessing (a) Original RGB image, reproduced with permission from CASIA dataset, (b) YCbCr image, (c) Separating Y, Cb and Cr bands, (b)(c) Image is generated by our method.

**Fig 7:**
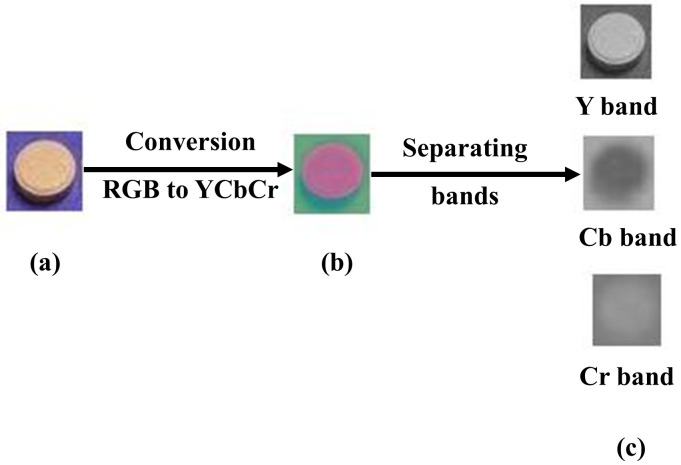
Patch preprocessing (a) Patch RGB image (b) YCbCr patch image (c) Separating Y, Cb and Cr bands, (a)(b)(c) Images are generated by our method.

In this process, both the original Image *I*_o_ and patch images *I*_p_ are converted from the RGB to YCbCr color domain to allow for easier manipulation in the following steps visually shown in Fig 6 and Fig 7. Where Y_o_, Cb_o_ and Cr_o_ are the original image sub bands and Y_p_, Cb_p_, Cr_p_ are the patch image sub bands. Y(luminance) represents the intensity or brightness), Cb (chrominance blue) represents the blue difference chroma component, Cr (chrominance red) represents the red difference chroma component.

The formal mathematical representation of the transformation from RGB to YCbCr color space as shown in Fig 6.

### 3.2 Discrete wavelet transformation

**Fig 8:**
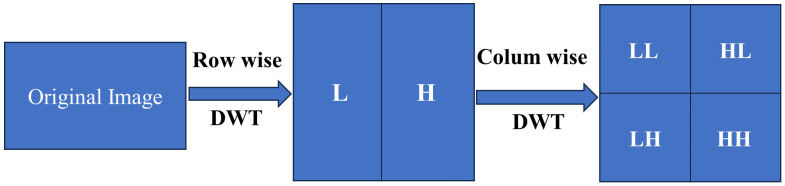
First level of wavelet decomposition.

Our objective is to execute forgery in utilizing wavelet transforms. The two-dimensional wavelet transforms at level one using the Haar wavelet is a method to decompose an image into low (L) and high (H) frequency sub bands. After this horizontal transformation, the left portion of the image contains the low-frequency information from each row, and the right portion holds the high-frequency details. Colum wise vertical transformations result into four sub bands as shown in Fig 8.

**Fig 9:**
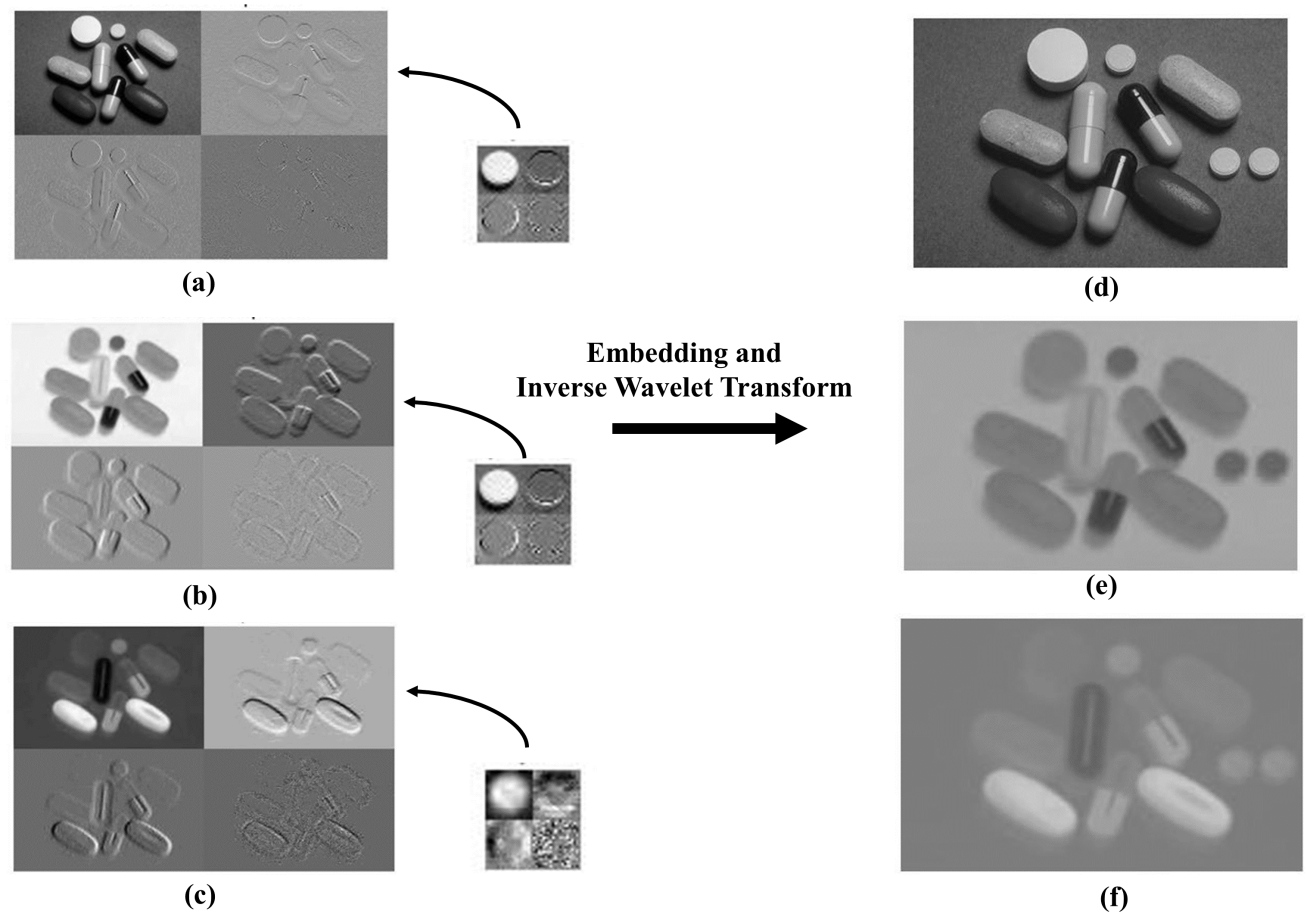
Applying Wavelet Transform (a), (b), (c) Wavelet processed original and patch image Y, Cb and Cr bands. (d), (e), (f) Inverse Wavelet transform of processed image Y, Cb and Cr bands, Image is generated by our method.


Here wavelet transform is applied on each band of the YCbCr original and patch images as in Fig 9(a), Fig 9(b) and Fig 9(c). Discrete Wavelet Transform (DWT) at level 1 is applied to each Y, Cb, and Cr component of images ***I***_***o***_, and ***I***_***p***_ producing four sub-bands per component (LL, HL, LH, and HH). The LL sub-band, which contains the majority of the image’s energy, is a low-pass version of the image at half its original size, while HL, LH, and HH represent the high-frequency information in horizontal, vertical, and diagonal directions, respectively. Wavelet transforms are characterized by properties like symmetry, smoothness, filter length compactness, and orthogonality. In this case, the Haar wavelet is chosen for simplicity. The wavelet process sub bands of original image in Y, Cb, Cr channels mathematically are shown in equations (1), (2), (3).

$$\begin{array}{c} \left\{{LL}_{yo},\;{HL}_{yo},\;{LH}_{yo},\;{HH}_{yo}\right\}=\;DWT\left(Y_{o}\right) \end{array}$$ (1)

$$\begin{array}{c} \left\{{LL}_{cbo},\;{HL}_{cbo},\;{LH}_{cbo},\;{HH}_{cbo}\right\}=\;DWT\left({Cb}_{o}\right) \end{array}$$ (2)

$$\begin{array}{c} \left\{{LL}_{cro},\;{HL}_{cro},\;{LH}_{cro},\;{HH}_{cro}\right\}=\;DWT\left({Cr}_{o}\right) \end{array}$$ (3)

The wavelet processed sub bands of patch image are shown in equations (4), (5), (6).


$$\begin{array}{c} \left\{{LL}_{yp},\;{HL}_{yp},\;{LH}_{yp},\;{HH}_{yp}\right\}=\;DWT\left(Y_{p}\right) \end{array}$$ (4)

$$\begin{array}{c} \left\{{LL}_{cbp},\;{HL}_{cbp},\;{LH}_{cbp},\;{HH}_{cbp}\right\}=\;DWT\left({Cb}_{p}\right) \end{array}$$ (5)

$$\begin{array}{c} \left\{{LL}_{crp},\;{HL}_{crp},\;{LH}_{crp},{\;HH}_{crp}\right\}=\;DWT\left({Cr}_{p}\right) \end{array}$$ (6)

#### 3.2.1 Embedding of patch image into host image.

After decomposition of wavelet transform, the concatenation of the sub-bands of the patch region of different sizes with those of the host image on different locations is perform here to get the transformed multiple copy move forged image.

To embed the DWT coefficients of the cropped image (I_p_) of each band Y, Cb and Cr into the original image (I_o_) for each channel (Y, Cb, and Cr) as mathematically shown in following equations (7), (8), (9), (10).

Embedding of Patch image Y band ‘Y_p_’ into the original image Y band ‘Y_o_’ Where for all i=1,2.... X and j=1,2...Y.


$$\begin{array}{c} {LL}_{yo}\;(i+p,\;j+q)=\;{LL}_{yp}\;(i,\;j) \end{array}$$ (7)

$$\begin{array}{c} {HL}_{yo}\;(i+p,\;j+q)=\;{HL}_{yp}\;(i,\;j) \end{array}$$ (8)

$$\begin{array}{c} {LH}_{yo}\;(i+p,\;j+q)=\;{LH}_{yp}\;(i,\;j) \end{array}$$ (9)

$$\begin{array}{c} {HH}_{yo}\;(i+p,\;j+q)=\;{HH}_{yp}\;(i,\;j) \end{array}$$ (10)

Embedding of Patch image Cb band ‘Cb_p_’ into the original image Cb band ‘Cb_o_’ for all i=1,2...X and j= 1,2...Y in the following equation (11), (12), (13), (14).


$$\begin{array}{c} {LL}_{Cbo}\;(i+p,\;j+q)=\;{LL}_{Cbp}\;(i,\;j) \end{array}$$ (11)

$$\begin{array}{c} {HL}_{Cbo}\;(i+p,\;j+q)=\;{HL}_{Cbp}\;(i,\;j) \end{array}$$ (12)

$$\begin{array}{c} {LH}_{Cbo}\;(i+p,\;j+q)=\;{LH}_{Cbp}\;(i,\;j) \end{array}$$ (13)

$$\begin{array}{c} {HH}_{Cbo}\;(i+p,\;j+q)=\;{HH}_{Cbp}\;(i,\;j) \end{array}$$ (14)

Embedding of Patch image Cr band ‘Cr_p_’ into the original image Cr band ‘Cr_o_’ Where for all i=1, 2....X and j=1,2...Y as shown in the following equations (15), (16), (17), (18).


$$\begin{array}{c} {LL}_{Cro}\;(i+p,\;j+q)=\;{LL}_{Crp}\;(i,\;j) \end{array}$$ (15)

$$\begin{array}{c} {HL}_{Cro}\;(i+p,\;j+q)=\;{HL}_{Crp}\;(i,\;j) \end{array}$$ (16)

$$\begin{array}{c} {LH}_{Cro}\;(i+p,\;j+q)=\;{LH}_{Crp}\;(i,\;j) \end{array}$$ (17)

$$\begin{array}{c} {HH}_{Cro}\;(i+p,\;j+q)=\;{HH}_{Crp}\;(i,\;j) \end{array}$$ (18)

In the above equations p and q are the variables for the vertical and horizontal embedding positions in the original image and X and Y are the dimensions of the patch image.

#### 3.2.2 Inverse wavelet transforms.

Once the patch image is embedded on host image then the inverse wavelet transform is applied to construct the tampered Y, Cb, and Cr bands shown in Fig 9(d), Fig 9(e) and Fig 9(f).

Mathematically the inverse DWT is applied to the modified wavelet components in Y, Cb, and Cr bands represented shown in equation (19), (20), (21).


$$\begin{array}{c} Y_{o}^{\prime }\;=\;IDWT\;\left({LL}_{yo},{\;HL}_{yo},\;{LH}_{yo},\;{HH}_{yo}\right) \end{array}$$ (19)

$$\begin{array}{c} {Cb}_{o}^{\prime }\;=\;IDWT\;\left({LL}_{cbo},\;{HL}_{cbo},\;{LH}_{cbo},\;{HH}_{cbo}\right) \end{array}$$ (20)

$$\begin{array}{c} {Cr}_{o}^{\prime }\;=\;IDWT\;\left({LL}_{cro},\;{HL}_{cro},\;{LH}_{cro},\;{HH}_{cro}\right) \end{array}$$ (21)

Where *Y*^*’*^*o*, *Cb*^*’*^*o and Cr*^*’*^*o* is the forged image Y, Cb, Cr bands after embedding the patch image’s components of the DWT components into the original image’s DWT components.

### 3.3 Post processing

**Fig 10:**
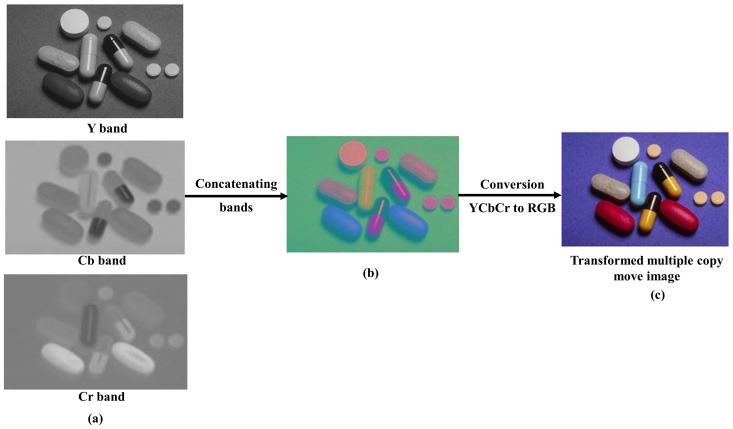
Post processing (a) Concatenation of Y, Cb and Cr band (b) YCbCr forged image (c) Conversion of YCbCr to RGB color domain, images are generated by our method.

The “tampered image *I*^*’*^_*YCbCr*_” is the final product of the YCbCr manipulation method. It is obtained by combining the Y_o_, Cb_o_, and Cr_o_ color channel to form a complete YCbCr image and follow the steps converted to the RGB image. The following Fig 10. illustrates the post processing operation and demonstrates the final image I’_RGB_ as output of a multiple transformed copy move forgery.

## 4. Experimental result

In this section the implementation of multiple transformed copy move forgery is performed. All implementation is accomplished in environment of MATLAB version 2018 on 64-bit Windows OS intel core i5 @2.5 and 8 GB of RAM. The method is designed to produce no tampering evidence and works well for similar and smooth texture backgrounds. It does not cause motion blur, edge inconsistencies, or region duplication as manual editing or other software tools might.

**Fig 11:**
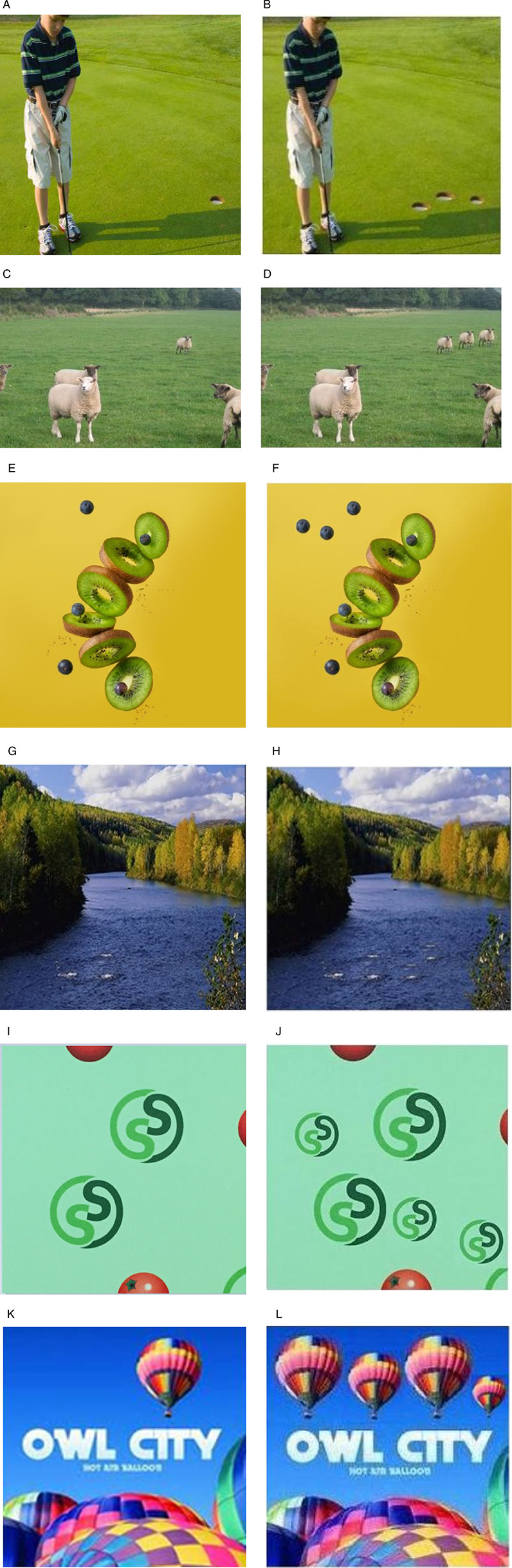
Example images of proposed method (a) Original Gulf image (b) Transformed multiple copy move gulf image with same patch size (c) Original animal image (d) Transformed multiple copy move animal image with same patch size (e) Original kiwi image (f) Transformed multiple copy move kiwi image with same patch size (g) Original lake image (h) Transformed multiple copy move image with different patch size (i) Original geometrical design image (j) Transformed multiple copy move geometrical design image with different patch size (k)Original balloon image (l) Transformed multiple copy move balloon image with different patch size. (a),(c),(g),(i) reproduced with permission from CASIA dataset, (b), (d), (f), (h), (j), (i) images are generated by our method. (k) reproduced with permission from http://www.famousfix.com/topic/owl-city-hot-air-balloon-album) (e) reproduced with permission from (https://unsplash.com/s/photos/kiwi-fruits).

We employed our approach on a set of images sourced from CASIA dataset and various online platforms. Six images are selected as shown in Fig 11. The kiwi image is free downloaded from (https://unsplash.com/s/photos/kiwi-fruits), balloon image from (http://www.famousfix.com/topic/owl-city-hot-air-balloon-album) and lake image, gulf image, geometrical design image, animal image, bird image is taken from CASIA dataset. These images are tested and compared with advanced methods from existing literature, the results proved to be interesting.

### 4.1 Evaluation metrics

The effectiveness of the forgery detection methods is usually gauged by two measures, detection accuracy (r) and false detection rate or FDR (w). These are computed by the equations (22) and (23).


$$\text{r}=\frac{\left|\text{R}\cap \text{D}\right|}{\left|\text{R}\right|}$$ (22)

$$\begin{array}{c} w=\frac{\left|F-D\right|}{\left|R\right|} \end{array}$$ (23)

where R represents the actual tampered area, D is a detected area, and F is a falsely detected area.

### 4.2 Benchmark methods

#### 4.2.1 Soumya’s method.

To assess the performance of proposed method, we compare our results with the result of Soumya’s method [9]. The Soumya’s method employs Stationary wavelet transform (SWT) to convert color-forged images into grayscale, generating LL sub-bands for feature extraction and analysis. AKAZE is utilize to collect key points from the LL sub-band, providing shift-invariant and rotation-invariant features for forgery detection. This proposed model utilizes DBSCAN for clustering similar features extracted by AKAZE, aiding in the identification of forged regions. A unique discrete uniform quantization method is computed to remove outliers in the shift distance matrix, enhancing the accuracy of forgery detection. Experimental results demonstrated on multiple forgery and different post processing attacks. Additionally, the method is evaluated for detecting tampered areas attributed to smooth regions, demonstrating the capability to identify forged regions accurately to a certain extent.

#### 4.2.2 Patrick’s method.

The method proposed in [10], is grounded on the identification of image blobs and the utilization of Binary Robust Invariant Scalable Key points (BRISK) feature. The procedure comprises several stages: firstly, the regions of interest, referred to as image blobs and BRISK feature, are detected within the input image; subsequently, the BRISK key points situated within the same blob are identified. Finally, the matching procedure is executed among BRISK key points located in distinct blobs to find similar key points for copy-move regions. The suggested method experiments show effectiveness in handling geometric transformations and have resilience to different post-processing attacks.

After covering the existing techniques from literature, the next section of this study presents our findings. It demonstrates with an illustrative simulation example of proposed method with different multiple forged region types, followed by an overview of the comprehensive results on state-of-the-art approaches.

### 4.3 Simulation results with examples

**Fig 12:**
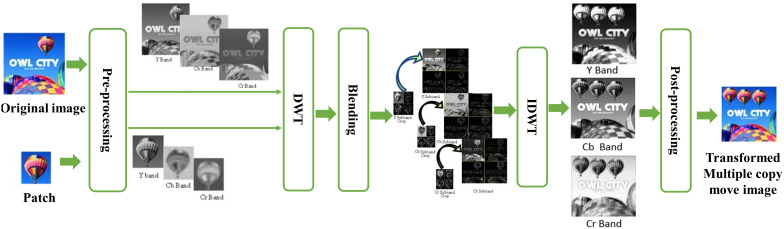
Transformed multiple copy move forgery, reproduced with permission from http://www.famousfix.com/topic/owl-city-hot-air-balloon-album).

The overall procedure for this specific manipulation is visually depicted in the following Fig 10. Initially, a small region (depicting a hot air balloon) was cropped from the original image, serving as a patch to be pasted at a predefined positions within the host image. Since we conducted a copy move manipulation, the original image also served as the host image. In Fig 10. both the patch and host were transformed into the YCbCr color space; subsequently, each resulting component underwent Discrete Wavelet Transform (DWT). For illustrative purposes, displays a single-level DWT (l = 1) application, resulting in four sub-bands for each of the three YCbCr components of both the host and the patch. Each sub-band of the patch was pasted onto its corresponding host sub-band of a given component, yielding the manipulated DWT domain Y, Cb, and Cr components, as illustrated in Fig 12. The subsequent application of inverse DWT produced the Y, Cb, and Cr components of the manipulated image. These three components were then merged to generate the final multiple forge RGB image showcased in following Fig 12.

**Fig 13:**
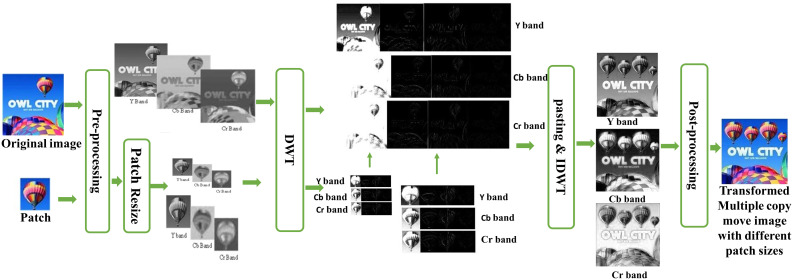
Multi region transformed copy move forgery with different patch sizes, reproduced with permission from http://www.famousfix.com/topic/owl-city-hot-air-balloon-album).

In Fig 13. Multi region copy move forgery with the resize patch image manipulation is visually depicted. In this proposed method the patched image undergoes resizing during the preprocessing phase, following its conversion into the YCbCR color channel. In subsequent steps, after applying the wavelet transform each patch is pasted on to the pre-define location in image. Rest of the figure proceeds the same steps as involved in the Fig 12. to get the transformed multiple copy move forgery with different patch sizes.

**Fig 14:**
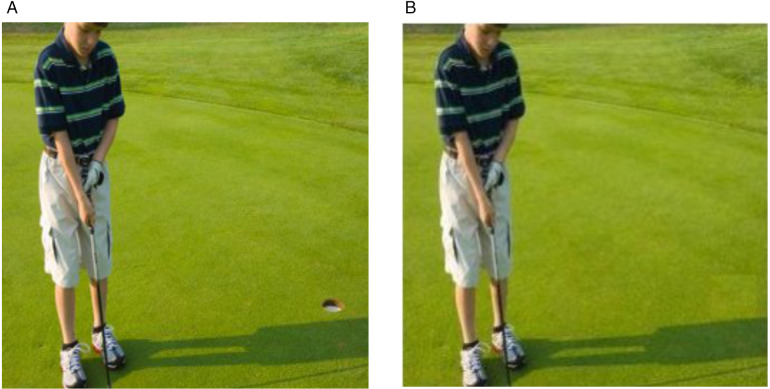
Copy move forgery with hiding objects (a) Original image, reproduced with permission from CASIA Dataset (b) Transformed copy move image, image is generated by our method.

Despite improvements in copy-move forgery detection, a significant gap remains in cases where an object in same image is replaced with the background to effectively hiding the objects as illustrated in Fig 14. This type of copy move forgery often goes undetected because techniques used in conventional and deep learning approaches, such as image segmentation and key point detection where primary focus is on identifying objects within the image [20, 21]. When objects are hide or replaced with background elements, these regions are not properly segmented and leading to a failure in detection accuracy. Therefore, further exploration is required to develop methods capable of addressing this type of copy move forgery.

### 4.4 Comparative analysis

The simulation results in section 4.3, it is challenging to detect manipulation visually particularly without the original reference. To provide a visual representation, outcomes of two additional approaches are displayed in Fig.12 and Fig.13 [9,10]. Nevertheless, subjective findings should not be the sole basis for assessment; hence, evaluating the efficacy of the proposed approach against established methodologies is essential. Consequently, our analysis leaned on two distinct techniques, i.e., “A copy‑move forgery detection technique using DBSCAN‑based key point similarity matching” in [9] and “Copy-move forgery detection using image blobs and BRISK feature” in [10]. Both techniques are based on key point features that are considered more effective for detecting forgeries under post processing attacks like rotation and scaling.

**Fig 15:**
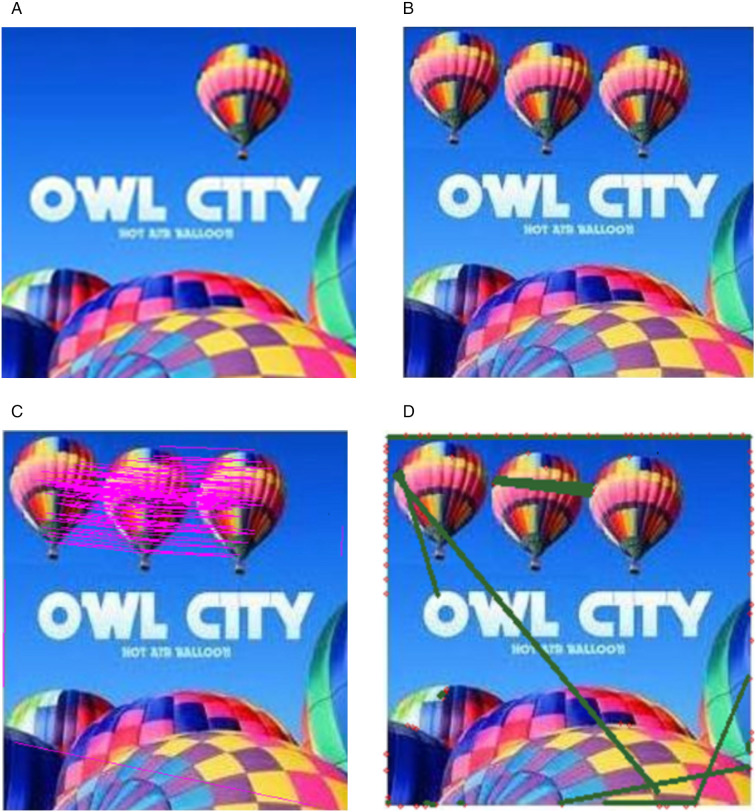
Detection Results (a) Original image (b) Multi Region Transformed multiple Copy move balloon image, (c) Soumya’s method detection results, (d) Patrick’s Method detection results. Original image reproduced with permission from the http://www.famousfix.com/topic/owl-city-hot-air-balloon-album) (b) image generated by our method, and (c), (d) show the detection results on (b).

The image shown in Fig 15. demonstrates multiple forged image results evaluated using existing state of art approaches. In Fig 15. (a) image is the forged image using the proposed method and Fig 15. (b) presents the detection results using Soumya’s method. I am grateful to the author for her valuable time and contributions, which have been instrumental in concluding my work. Although the results are satisfactory with the same patch size but the detection is not consistent in all cases. Fig 15.(c) depicts the results obtained using Patrick’s method, which is available on GitHub link (https://github.com/niyishakapatrick/Copy-move-forgery-detection-using-image-blobs-BRISK-features). This method was applied to the images processed by the proposed method, showing BRISK features represented by red dots and then similar features in form of red dots are mapped with the green lines. The method only identifies a few similar features and fails to precisely locate the forged regions.

**Fig 16:**
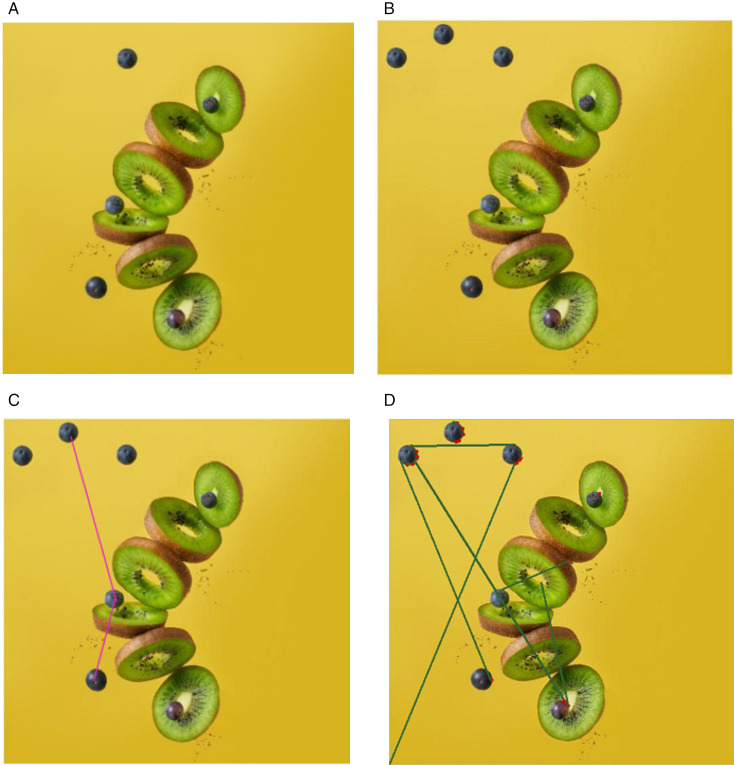
Detection results (a) Original image (b) Multi Region Transformed multiple copy move kiwi image, (c) Soumya’s method detection results, (d) Patrick’s Method detection results. Original image reproduced with permission from the (https://unsplash.com/s/photos/kiwi-fruits). (b) image generated by our method, and (c), (d) show the detection results on (b).

Another example, illustrated in Fig 16. demonstrates the detection results evaluated by both methods. The original kiwi image is referred to as the “original image,” while Fig 16. (a) presents the transformed multiple copy move image, where the patch size remains consistent. In Fig 16. (b), the detection results using Soumya’s method show zero accuracy and unsatisfactory outcomes. Conversely, Fig 16. (c) displays the results from Patrick’s method which also fails to accurately detect the similar points. The evaluation of both methods reveals significant limitations in accurately detecting transformed multiple copy move regions, highlighting the need for improved algorithms.

**Fig 17:**
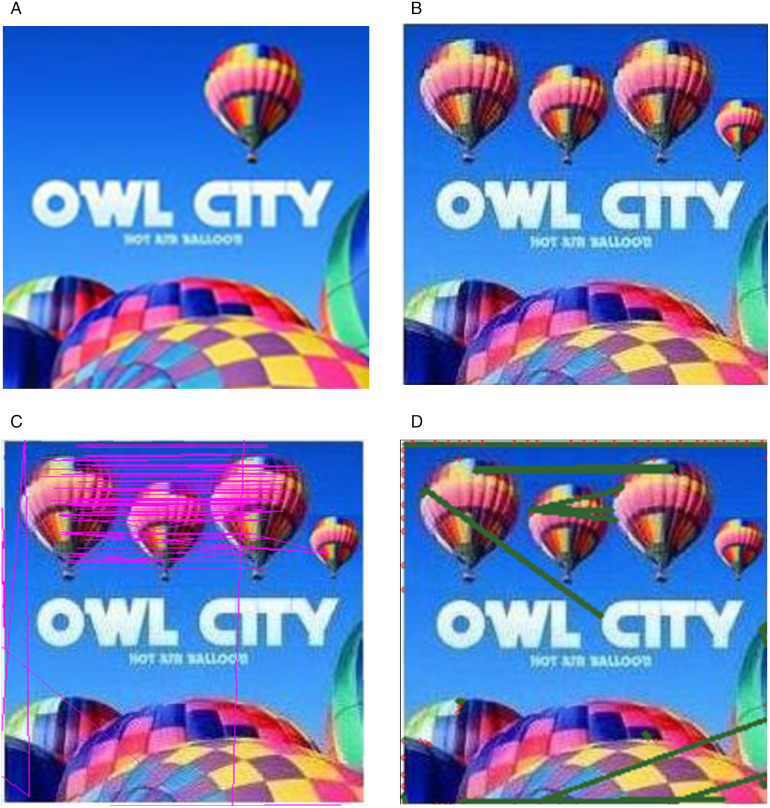
Detection results (a) Original image (b)Multi Region Transformed multiple copy move balloon image with different patch sizes, (c) Soumya’s method detection results, (d) Patrick’s Method detection results. Original image reproduced with permission from the (http://www.famousfix.com/topic/owl-city-hot-air-balloon-album). (b) image generated by our method, and (c), (d) show the detection results on (b).

In Fig 17. multiple regions of the balloon image are forged using different patch sizes, as illustrated in Fig 17(a). The image patch is transformed into three different sizes to create a multiple transformed forged image. This forged image is then evaluated using existing detection approaches. Fig 17(b) shows the results of Soumya’s method which can detect slight changes in region of interest, though smaller objects remain undetected. Conversely, in Fig 17. (c) Patrick’s method demonstrates unsatisfactory detection results in all cases with the no accuracy.

**Fig 18:**
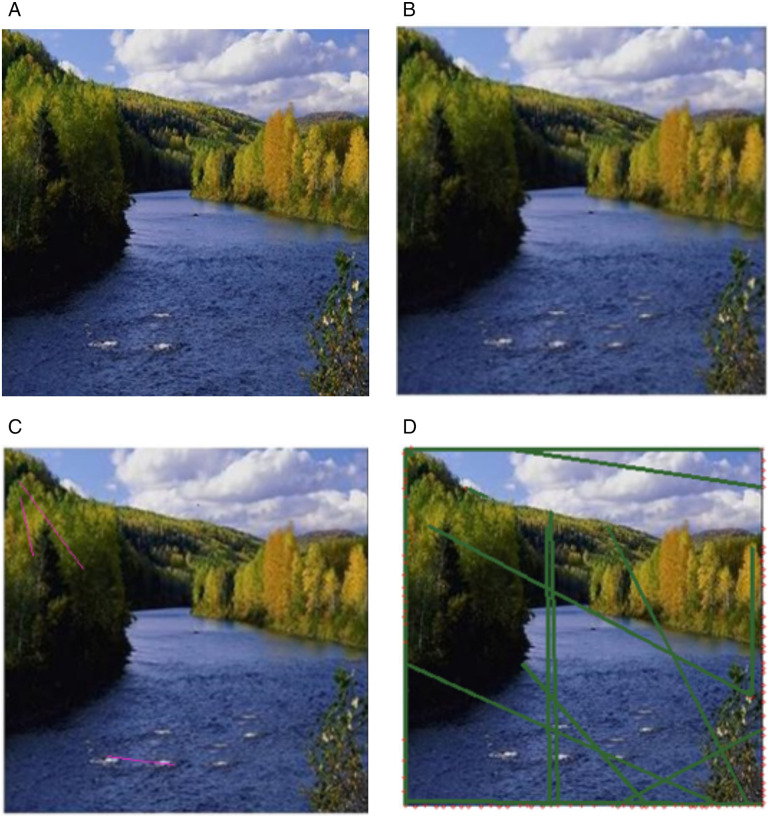
Detection results (a) Original image (b) Multi Region Transformed multiple copy move lake image with different patch sizes, (c) Soumya’s method detection results, (d) Patrick’s method detection results. Original image reproduced with permission from the CASIA dataset. (b) image generated by our method, and (c), (d) show the detection results on (b).

Similarly, another example is presented in Fig 18. featuring a lake image referred as the “original image”. Fig 18(a) displays the transformed multiple copy-move forged image with varying patch sizes. In Fig 18(b), the detection results using Soumya’s method are shown, which prove to be unsatisfactory. Fig 18(c) presents the results of Patrick’s method, which detects natural similarities but fails to accurately identify the region of interest.

## 5. Conclusion

Through the manipulation of images in the frequency domain, followed by the application of the DWT, we achieved consistent and reliable outcomes. The assumption that the DWT possesses sufficient capability to eliminate any artifacts or unintended consequences arising from manipulation was confirmed, particularly in the case of multiple copy move forgeries. However, our experiments revealed that the state of art detection methods were not particularly effective in pinpointing the multiple copy move forged areas. Consequently, there is a need to enhance and fine-tune the forgery detection techniques that may exhibit the properties of transformed multiple copy move forgery detection as well as the ability to identify the resized patch images.

In future work regarding forgeries, it has been observed that most experiments on multiple copy-move forgery in the transformed domain are typically conducted on images with uniform and smooth textured backgrounds as in Fig 15 and Fig 16. However, when applied to images with highly textured regions, the wavelet transforms ineffectively conceal the patch image edges. To address this limitation, future research should find the use of alternative image processing techniques which may provide better performance in highly texture regions and enhance the robustness of forgery detection in more complex image scenarios.

## References

[1] Schetinger V, Iuliani M, Piva A, Oliveira MM. Digital image forensics vs. image composition: an indirect arms race. arXiv. 2016. https://arxiv.org/abs/1601.03239

[2] Qazi T, Hayat K, Khan SU, Madani SA, Khan IA, Kołodziej J, et al. Survey on blind image forgery detection. IET Image Processing. 2013;7(7):660–70. doi: 10.1049/iet-ipr.2012.0388

[3] Qazi T, Ali M, Hayat K, Magnier B. Seamless copy-move replication in digital images. J Imaging. 2022;8(3):69. doi: 10.3390/jimaging8030069 35324624 PMC8954403

[4] Jaiswal AK, Srivastava R. Detection of copy-move forgery in digital image using multi-scale, multi-stage deep learning model. Neural Process Lett. 2021;54(1):75–100. doi: 10.1007/s11063-021-10620-9

[5] Agarwal R, Verma O. An efficient copy-move forgery detection using deep learning feature extraction and matching algorithm. Multimedia Tools and Appl. 2020.

[6] Wang Y, Kang X, Chen Y. Robust and accurate detection of image copy-move forgery using PCET-SVD and histogram of block similarity measures. J Info Security Appli. 2020;54:102536. doi: 10.1016/j.jisa.2020.102536

[7] Farhan MH, Shaker K, Al-Janabi S. Copy–move forgery detection in digital image forensics: A survey. Multimed Tools Appl. 2024.

[8] Verma M, Singh D. Survey on image copy-move forgery detection. Multimed Tools Appl. 2023;83(8):23761–97. doi: 10.1007/s11042-023-16455-x

[9] Mukherjee S, Pal AK, Maji S. A copy-move forgery detection technique using DBSCAN-based key point similarity matching. Int J Mach Learn & Cyber. 2024.

[10] Niyishaka P, Bhagvati C. Copy-move forgery detection using image blobs and BRISK feature. Multimed Tools Appl. 2020;79(35–36):26045–59. doi: 10.1007/s11042-020-09225-6

[11] Hayat K, Qazi T. Forgery detection in digital images via discrete wavelet and discrete cosine transforms. Comput Electr Eng. 2017;62:448–58.

[12] Ng T, Chang S, Lin C, Sun Q. Passive-blind Image Forensics. In Multimedia Security Technologies for Digital Rights. Elsvier:Amsterdam, The Netherlands, 2006.

[13] Sharma D, Abrol P. Digital image tampering—A threat to security management. Int J Adv Res Comput Commun Eng. 2013;2:4120–3.

[14] Walia S, Saluja K. Digital image forgery detection: a systematic scrutiny. Aus Jf Forensic Sci. 2018.

[15] Zedan I, Soliman M, Elsayed K, Onsi H. Copy move forgery detection techniques: a comprehensive survey of challenges and future directions. Int J Adv Comp Sci Appl. 2021.

[16] Wang X, Wang C, Wang L, Yang H, Niu P. Robust and effective multiple copy-move forgeries detection and localization. Pattern Anal Applic. 2021;24(3):1025–46. doi: 10.1007/s10044-021-00968-y

[17] Meena KB, Tyagi V. A hybrid copy-move image forgery detection technique based on Fourier-Mellin and scale invariant feature transforms. Multimedia Tools Appl. 2020.

[18] Meena KB, Tyagi V. A copy-move image forgery detection technique based on tetrolet transform. J Info Security Appl. 2020;52:102481. doi: 10.1016/j.jisa.2020.102481

[19] Aria M, Hashemzadeh M, Farajzadeh N. QDL-CMFD: A Quality-independent and deep Learning-based Copy-Move image forgery detection method. Neurocomputing. 2022;511:213–36. doi: 10.1016/j.neucom.2022.09.017

[20] Gul F, Shah M, Ali M, Qazi T, Ahmad M, Mehmood A. An effective adaptive downsampling method for high-resolution multi-panel image segmentation. IEEE Access. 2025;13:44872–83. doi: 10.1109/access.2025.3548431

[21] Gul F, Shah M, Ali M, Hussain L, Sadiq T, Abbasi AA, et al. A hybrid multi-panel image segmentation framework for improved medical image retrieval system. PLoS One. 2025;20(2):e0315823. doi: 10.1371/journal.pone.0315823 39977397 PMC11841867

